# 

**Published:** 1910-04-02

**Authors:** 


### THE HOSPITAL

Telegraphic Address: Ospedale, London.

THE MODERN MEDICAL AND INSTITUTIONAL JOURNAL.

Telephone Number: 2734 Gerrard.

NEW SERIES. No. 163, Vol. VII.

No. 1235, Vol. XLVIII.

SATURDAY, APRIL 2ND, 1910.

PRICE THREEPENCE

